# Integrated Analysis of Transcriptomics and Proteomics Provides Insights into the Accumulation Mechanism of Ascorbic Acid in *Rosa roxburghii* Tratt

**DOI:** 10.3390/foods14050748

**Published:** 2025-02-22

**Authors:** Pei Li, Bo Mu, Jing Liu, Wenqing Wu, Can He, Boxi Tan, Shijing Tang, Lu Yu

**Affiliations:** 1 Guizhou Key Laboratory of Miao Medicine, Qiandongnan Engineering and Technology Research Center for Comprehensive Utilization of National Medicine, Kaili University, Kaili 556011, China; 2 School of Liquor and Food Engineering, Guizhou University, Guiyang 550025, China; lily55ljing@163.com (J.L.); wuwenqing0809@163.com (W.W.); 18212045070@163.com (C.H.); 18090952291@163.com (B.T.); 17585204647@163.com (S.T.); 3 Guizhou Academy of Testing and Analysis, Guiyang 550000, China; 18143522730@163.com

**Keywords:** *Rosa roxburghii* Tratt, AsA accumulation, ascorbate and aldarate metabolism, transcriptomic, proteomic

## Abstract

*Rosa roxburghii* Tratt (RRT) is widely cultivated in Guizhou Province, China. In recent years, RRT has emerged as one of the most promising new fruit crops in China, primarily because of its remarkably high levels of ascorbic acid (AsA). In this research, we assessed the AsA levels in RRT across various growth phases. The findings demonstrate that the AsA concentration in RRT fruits progressively increased in a linear fashion throughout development, peaking at 2274.60 mg/(100 g FW) when the fruit reached maturity (84 DAA). Furthermore, we conducted an integrated analysis of transcriptomic and proteomic data for the first time to investigate the mechanisms responsible for AsA accumulation in RRT. Our results show that differentially expressed genes (DEGs) and differentially abundant proteins (DAPs) were primarily associated with the ascorbate and aldarate metabolism pathway, suggesting that this pathway plays a crucial role in regulating AsA accumulation in RRT. This study elucidates the molecular mechanism underlying AsA accumulation in RRT and provides a robust scientific foundation for subsequent research on AsA accumulation in RRT.

## 1. Introduction

*Rosa roxburghii* Tratt (RRT), commonly known as the Chestnut rose, belongs to the rose family and is a wild fruit species native to southwestern China, with extensive cultivation particularly prominent in Guizhou Province [1]. Recently, RRT has been recognized as one of China’s three promising new fruit crops, owing to its rich nutritional and medicinal properties [2]. These include high levels of ascorbic acid (AsA), superoxide dismutase (SOD), flavonoids, polysaccharides, amino acids, organic acids, and mineral elements [1,2,3]. Additionally, RRT offers various health benefits, such as enhancing immune function, exhibiting anti-atherosclerosis properties, delaying aging, preventing cancer, lowering blood pressure, scavenging free radicals, and mitigating lipid peroxidation damage [4,5]. Particularly, RRT, often referred to as the “King of vitamin C”, is distinguished by its exceptionally high levels of AsA, with its fruits containing more than 1100 mg/(100 g fresh weight (FW)) and its leaves containing over 160 mg/(100 g FW) [3,6].

AsA, also named as vitamin C, is among the most prevalent water-soluble antioxidants found in both plants and animals. This compound is essential for human health and serves a critical function in defending against diseases associated with collagen synthesis and oxidative stress [7]. In plants, AsA functions as a key redox buffer and plays a crucial role in regulating numerous physiological processes that govern growth, development, signal transduction, and stress resilience [8,9,10]. Recent research into the biosynthesis, oxidation, recycling, transport, and redox regulation of AsA has significantly expanded our understanding of the mechanisms underlying AsA’s influence on plant growth, development, and stress tolerance [11,12,13,14,15,16]. Given that plant-based foods are the primary dietary source of AsA for humans, there has been growing interest in enhancing the AsA content of plants to boost their nutritional quality [17]. The interplay between reactive oxygen species (ROS) and AsA in modulating cell signaling and metabolic pathways is now well recognized [18]. Unlike animals, which rely on a single pathway for AsA biosynthesis, plants employ multiple pathways, highlighting their metabolic diversity and the critical role of AsA in plant health [19]. Alterations in cellular AsA levels can have wide-ranging impacts on plant growth, development, and stress tolerance, as AsA is integral to redox signaling, cell cycle control, enzyme activity, and the expression of defense and stress-related genes [20]. Therefore, sufficient consumption of AsA through food is essential for maintaining normal physiological functions, with fruits and vegetables serving as the most abundant natural sources of AsA in the human diet. While the physiological functions of AsA have been extensively studied, the mechanisms governing AsA accumulation in RRT remain largely unexplored.

In the present study, the AsA contents in RRT fruits were investigated using the 2,6-dichloroindophenol (2,6-D) solution titration method across different growth periods, and then the underlying mechanisms of AsA accumulation in RRT fruits were elucidated for the first time using an integrated analysis of transcriptomic and proteomic techniques.

## 2. Materials and Methods

### 2.1. Sample Collection

*Rosa roxburghii* Tratt (RRT) (variety: Guinong 9) fruits were all collected from the fruit germplasm repository of Guizhou Hongcai Investment Group Co., Ltd. in Panzhou City, Guizhou Province, China. A total of 12 batches of RRT fruits (5 replicates for each batch, 100 g for each replicate) were collected from 28 May 2021 (7 days after anthesis, DAA) to 13 August 2021 (84 DAA), and each collection interval was 7 days. The RRT fruits were promptly frozen using liquid nitrogen and stored at −80 °C until they were needed.

### 2.2. AsA Content Determination

The ascorbic acid (AsA) concentrations in RRT fruits were performed by the using the 2,6-D solution titration method in GB 5009.86-2016 (Chinese standard method) with some modifications [21]. Briefly, the RRT fruits were promptly blended with 2% metaphosphoric acid. Subsequently, 20 g of this viscous mixture was transferred to a 100 mL volumetric flask containing 2% oxalic acid and diluted to the mark with distilled water. After thorough mixing, the solution was centrifuged at 6000× *g* for 10 min. A 10 mL aliquot of the resulting supernatant was then titrated with 2,6-dichloroindophenol until a pink hue appeared in the solution. The AsA content was calculated using the following Equation (1), where X (mg/(100 g FW)) represents the Vc content per 100 g of sample, T (mg/mL) is the concentration of the AsA standard solution equivalent to 1 mL of dye solution, V (mL) is the volume of dye solution used for titrating the filtrate, V_0_ (mL) is the volume of dye solution used for titrating the control, and W (g) is the fresh weight of the sample in the tested filtrate.

(1) $$X=\frac{(V-V_{0})\times T}{W}\times 100$$

### 2.3. Transcriptome Analysis

#### 2.3.1. RNA-Seq Library Construction and Sequencing

Total RNAs of RRT fruits (5 replicates for each batch, 10 g for each replicate), collected on 23 July 2021 (63 DAA, labeled as **A**) and 13 August 2021 (84 DAA, labeled as **C**), respectively, were extracted using RNAiso Plus Total RNA extraction reagent (TaKaRa, Tokyo, Japan) according to the manufacturer’s instructions [22]. The RNA quantity and purity were assessed using an ASP-3700 Micro-volume UV/Vis spectrophotometer (Avans-Biotechnology, Taipei, China). All samples exhibited OD_260_/OD_280_ ratios within the range of 1.8 to 2.0. Following the manufacturer’s protocol [23], 1 μg of total RNA was utilized for the synthesis of the first-strand cDNA using the TransScript One-Step gDNA Removal and cDNA Synthesis SuperMix (TRANS, Beijing, China).

Poly(A)^+^ mRNA was extracted from the pooled total RNA samples using Oligo(dT) magnetic beads. This mRNA was subsequently broken down into smaller fragments through a fragmentation buffer treatment. Using the Superscript Double-Stranded cDNA Synthesis kit (Invitrogen, Carlsbad, CA, USA), double-stranded cDNA was synthesized from the purified poly(A)^+^ mRNA. Following synthesis, the double-stranded cDNA underwent end-repair, phosphorylation, and addition of an “A” base in accordance with Illumina’s library preparation guidelines. The 3′-adenylated cDNA fragments were then ligated to Illumina paired-end adapters at their ends. To select suitable templates for subsequent enrichment, the ligation products were purified via 2% agarose gel electrophoresis. AMPure XP beads were used to screen cDNA fragments sized approximately 250–300 bp. Post end-repair and adapter ligation, PCR amplification was performed on the products, followed by purification using the QIAquick PCR Purification Kit (Qiagen, Valencia, CA, USA). The cDNA library was quantified with a Qubit2.0 Fluorometer (Invitrogen, CA, USA), and its concentration was adjusted to 1.5 ng/μL. The insert size of the cDNA library was confirmed using an Agilent Technologies 2100 bioanalyzer (Agilent Technologies, Palo Alto, CA, USA) [24]. Transcriptome sequencing (2 biological replicates for each sample) for samples **A** and **C** was carried out on the Illumina HiSeq™ 2000 platform (Illumina Inc., CA, USA) [25].

#### 2.3.2. Sequence Database Searching and Differentially Expressed Genes (DEGs) Identification

To enhance the quality of the sequencing data, reads with low quality, those containing adapters, or those with an unknown base percentage exceeding 10% were filtered out from the initial dataset. Subsequently, the quality metrics of the remaining reads, including Q20, Q30, and GC content, were assessed [26]. To ensure the reliability of downstream analyses, raw reads were assembled into high-quality transcripts using Trinity, a software tool specifically tailored for transcriptome assembly [27]. The orientation of the unigenes was determined by aligning the predicted genes against various reference databases, such as Nt, Nr, Swiss-Prot, Pfam, KOG, KEGG, and GO. For mapping the clean reads, Bowtie2 (version 2.2.5) was utilized to align them to reference genes, while HISAT was employed for genome alignment [28,29]. DEGs were identified using the DESeq R package (version 1.10.1) with a threshold of nominal *p*-value < 0.05 [30].

### 2.4. Proteomics Analysis

#### 2.4.1. Protein Extraction and LC-MS/MS Analysis

RRT fruits (5 replicates for each batch, 10 g for each replicate), collected on July 23rd, 2021 (63 DAA, labeled as **A**) and 13 August 2021 (84 DAA, labeled as **C**), respectively, were used as the materials for proteomics analysis. In each experiment, about 1.5 g of RRT fruit tissues were homogenized in liquid nitrogen. The fine powder obtained from RRT fruit tissues was suspended in a solution containing 10 mL of acetone with 0.015 g polyvinylpyrrolidone (PVPP), 10% trichloroacetic acid (TCA), and 0.07% β-mercaptoethanol, and then was stored overnight at −20 °C. Following this, the mixture was subjected to centrifugation at 8000× *g* for 30 min at 4 °C. The resulting precipitate was collected and washed three times with 10 mL of precooled acetone to remove TCA. Subsequently, the precipitate was resuspended in 5 mL of an ice-cold protein extraction buffer composed of 0.5 M Tris-HCl (pH 7.5), 0.7 M sucrose, 0.1 M KCl, 50 mM EDTA, and 40 mM dithiothreitol (DTT). Afterward, the protein solution was isolated by centrifugation at 12,000× *g* for 15 min at 4 °C. The samples were then enzymatically digested with trypsin in a buffer of 10 mM ammonium bicarbonate at 37 °C overnight. The peptides generated from trypsin digestion were extracted using equal volumes of a 60% acetonitrile (ACN) and 5% formic acid (FA) solution, dried under vacuum centrifugation, and finally reconstituted in 50 μL of HPLC-grade water containing 0.1% FA for LC−MS/MS analysis [31]. Crude peptides were purified by desalting on a ChromXP Trap column (Nano LC TRAP Column, 3 μm C18-CL, 120 Å, 350 μm × 0.5 mm; Foster City, CA, USA). They were subsequently separated on an analytical column, specifically a Nano LC C18 reversed-phase column (3 μm C18-CL, 75 μm × 15 cm, Foster City, CA, USA), using a linear gradient of mobile phases A (consisting of 5% ACN and 0.1% FA) and B (comprising 95% ACN and 0.1% FA) over a period of 120 min at a flow rate of 300 nL/min. To ensure the reliability of the results, the experiment was performed in triplicate with biological replicates.

#### 2.4.2. Sequence Database Searching and Differentially Abundant Proteins (DAPs) Identification

Wiff files of the sequence database were processed and quantified using ProteinPilotTM Software (v5.0.2). The MS/MS spectra were compared against the previously mentioned transcriptome-derived database. The precursor and fragment masses were identified with initial mass tolerances of 6 and 20 ppm, respectively. The search parameters included variable modifications such as methionine oxidation and *N*-terminal acetylation, along with a fixed modification for carbamidomethyl cysteine. The minimum peptide length was established at 7 amino acids, allowing for up to 2 missed cleavages. The MS runs were evaluated using the ‘match between runs’ feature, with a retention time window set to 20 s for matching purposes. Proteins that aligned with the reverse database were excluded from the results. To ensure the accuracy of both peptide and protein identifications, the iBAQ algorithm was used to rank the absolute abundance of different proteins within a single sample, and then the iBAQ data were used for Student’s *t*-test [31]. DAPs was defined with an expression level exceeding 1.5-fold and a *p*-value less than 0.05 for the *t*-test [31].

### 2.5. Bioinformatics Analysis of DEGs and DAPs

Gene Ontology (GO) annotations, which encompass biological processes (BP), cellular components (CC), and molecular function (MF), as well as Kyoto Encyclopedia of Genes and Genomes (KEGG) pathway enrichment analyses for the DEGs and DAPs, were conducted using the resources available at http://www.geneontology.org/ and https://www.kegg.jp/kegg/, respectively (accessed on 5 November 2021) [31].

### 2.6. Statistical Analysis

Mean values and standard deviation (SD) of AsA concentrations in RRT fruits across various growth phases were analyzed using the SPSS 17.0 software (SPSS Inc., Chicago, IL, USA).

## 3. Results

### 3.1. Ascorbic Acid (AsA) Content Assays

**Figure 1:**
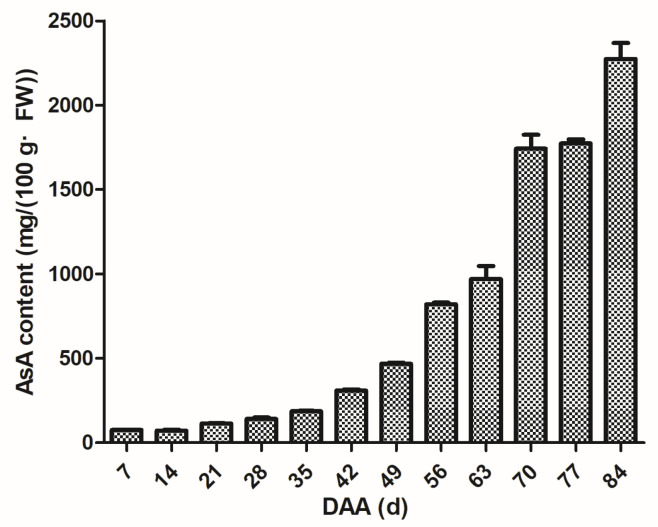
Changes in the ascorbic acid (AsA) contents in RRT in different growth periods. Vertical bars refer to mean ± standard deviation (SD) (*n* = 3).

The content of AsA in the RRT fruits, as shown in Figure 1, showed a linear growth trend throughout the development process. In the early stage of fruit development (7–42 DAA), the AsA content in the RRT fruits increases relatively slowly, and the average concentration is 155.85 mg/(100 g FW). Meanwhile, in the middle and late periods of fruit development (49–84 DAA), the AsA content in RRT fruits shows a trend in rapid growth, of which 49–70 DAA is the stage with the highest accumulation rate and the AsA content increases to 1274.69 mg/(100 g FW), which accumulated to more than half of the total content. When the fruit was ripe (84 DAA), the AsA content reached the highest level, with a value of up to 2274.60 mg/(100 g FW).

### 3.2. Quality Check of Transcriptome Sequencing Data

The total cDNA library prepared from RRT fruits was sequenced using Illumina HiSeq™ 2000 (Illumina Inc., San Diego, CA, USA), and the results are listed in Table S1. Table S1 shows that 47,491,254, 54,447,622 and 43,447,172, 43,119,412 raw reads (188,505,460 in total) were sequenced in samples **A** (collected on 63 DAA) and **C** (collected on 84 DAA), respectively. Table 1 shows that, after cleaning and quality checking, 46,202,096, 52,816,246, 42,425,254, and 41,712,142 clean reads (183,155,738 in total), with Q20 bases contents ranging from 97.62% to 97.80%, Q30 bases contents ranging from 93.29% to 93.68%, and GC contents ranging from 46.18% to 46.28%, were generated from the cDNA libraries. Meanwhile, as shown in Table 2, 108,211 transcripts, with the interval length ranging from 301 to 500 bp (18,279 transcripts), ranging from 501 to 1000 bp (24,922 transcripts), ranging from 1001 to 2000 bp (33,876 transcripts), and more than 2000 bp (31,134 transcripts) were assembled, and 36,342 unigenes were obtained with the average length of 1435 bp. In general, the sequencing results are of good quality, and the data can be used for subsequent bioinformatics analyses.

### 3.3. Protein-Coding Region (CDS) Prediction of Transcriptome Sequences

Table 3 shows that 36,342 unigenes were annotated based on the seven public databases. Among them, 21,857 (60.14%), 18,704 (51.46%), 17,322 (47.66%), 17,135 (47.14%), 5633 (15.49%), 7905 (21.75%), and 17,135 (47.14%) unigenes could be annotated with reference to the Nt, Nr, Swiss-Prot, Pfam, KOG, KEGG, and GO databases, respectively. Meanwhile, 2658 (7.31%) unigenes were annotated in all databases and 26,412 (72.67%) unigenes were annotated in at least one database.

### 3.4. DEGs Identification and Bioinformatics Analysis

**Figure 2:**
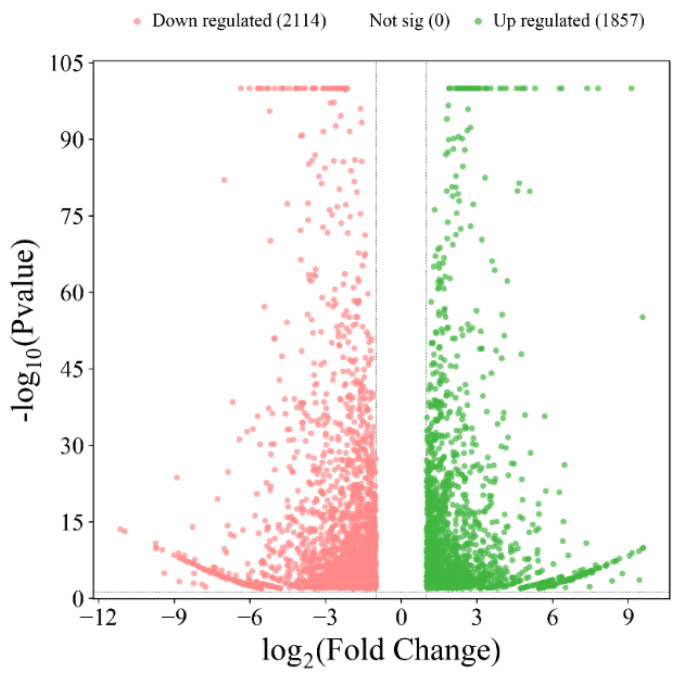
Volcano plot diagram of differentially expressed genes (DEGs) at **C** vs. **A**. The green points are significant up-regulated genes, while the red points are significant down-regulated genes.

When comparing sample **A** with **C**, a total of 3971 DEGs were detected (as shown in Figure 2 and Table S2), of which 1857 genes were up-regulated and 2114 genes were down-regulated.

**Figure 3:**
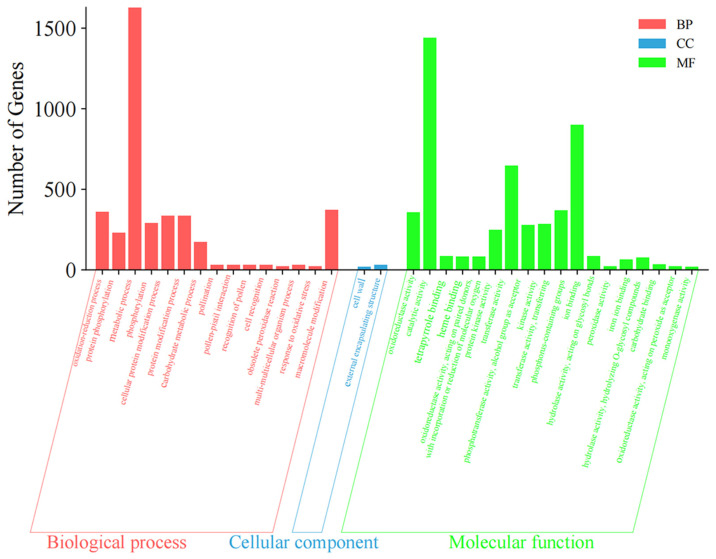
Gene Ontology (GO) term enrichment analysis of differentially expressed genes (DEGs) of **C** vs. **A**.

The GO term enrichment analysis of **C** vs. **A** (Figure 3 and Table S3) demonstrated that the main BP involved the oxidation reduction process, protein phosphorylation, metabolic process, phosphorylation, cellular protein modification process, protein modification process, carbohydrate metabolic process, pollination pollen–pistil interaction, recognition of pollen, cell recognition, obsolete peroxidase reaction, multi-multicellular organism process, response to oxidative stress, and macromolecule modification. The main CC involved the cell wall and an external encapsulating structure. The main MF involved oxidoreductase activity, catalytic activity, tetrapyrrole binding, heme binding, oxidoreductase activity, acting on paired donors, with incorporation or reduction in molecular oxygen, protein kinase activity, transferase activity, phosphotransferase activity, the alcohol group as an acceptor, kinase activity, transferase activity, transferring phosphorus-containing groups, ion binding, hydrolase activity, acting on glycosyl bonds, peroxidase activity, iron ion binding, and hydrolase activity, hydrolyzing *O*-glycosyl compounds, carbohydrate binding, oxidoreductase activity, acting on peroxide as an acceptor, and monooxygenase activity.

**Figure 4:**
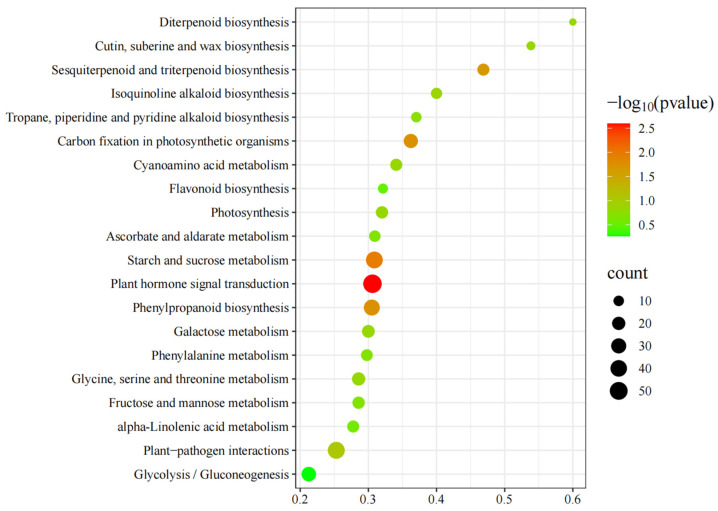
Top twenty Kyoto Encyclopedia of Genes and Genomes (KEGG) pathway enrichments of differentially expressed genes (DEGs) of **C** vs. **A**.

To further the functional characterization of the DEGs of **C** vs. **A**, pathway analysis based on the KEGG database was classified and annotated into 104 known KEGG pathways (Table S4). The KEGG pathway analysis of **C** vs. **A** (Figure 4 and Table S4) revealed that DEGs were mainly annotated into Plant hormone signal transduction, Starch and sucrose metabolism, Carbon fixation in photosynthetic organisms, Phenylpropanoid biosynthesis, Sesquiterpenoid and triterpenoid biosynthesis, Plant–pathogen interactions, Isoquinoline alkaloid biosynthesis, Cyanoamino acid metabolism, Cutin, suberine and wax biosynthesis, Photosynthesis, Diterpenoid biosynthesis, Galactose metabolism, Glycine, serine and threonine metabolism, Tropane, piperidine and pyridine alkaloid biosynthesis, Ascorbate and aldarate metabolism, Fructose and mannose metabolism, Phenylalanine metabolism, alpha-Linolenic acid metabolism, and Flavonoid biosynthesis.

### 3.5. DAPs Identification and Bioinformatics Analysis

**Figure 5:**
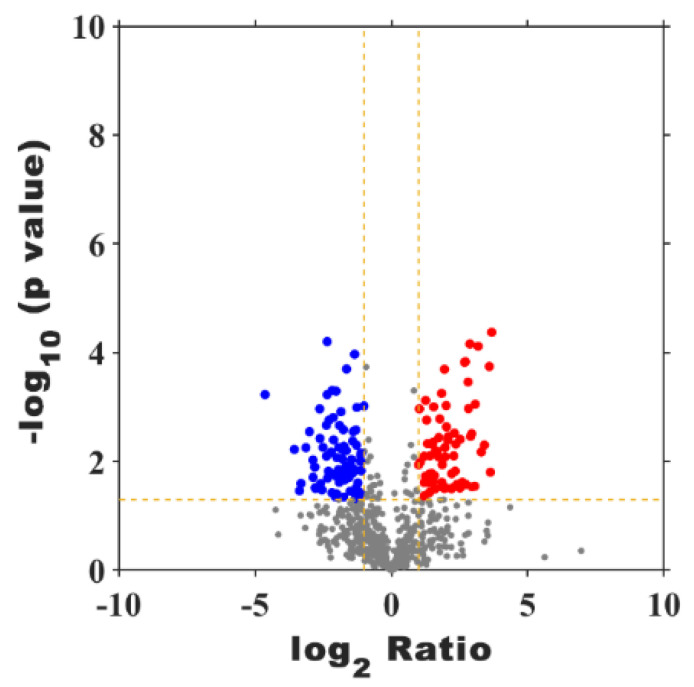
Volcano plot diagram of differentially abundant proteins (DAPs) at **C** vs. **A**. The red points are significant up-regulated proteins, the blue points are significant down-regulated proteins, while the grey dots are not significant changed proteins.

As illustrated in Table S5, a total of 1233 proteins were identified in both samples **A** and **C**, whereas 961 (78%) and 849 (69%) proteins were identified in samples **A** and **C**, respectively. Among these proteins, 656 (53%) proteins were common in both groups, whereas 305 (25%) and 193 (29%) proteins were uniquely expressed in samples **A** and **C**, respectively. Meanwhile, to identify DAPs, a volcano plot was utilized. As illustrated in Figure 5, when comparing sample **C** to **A**, there were 68 up-regulated and 83 down-regulated proteins, respectively.

**Figure 6:**
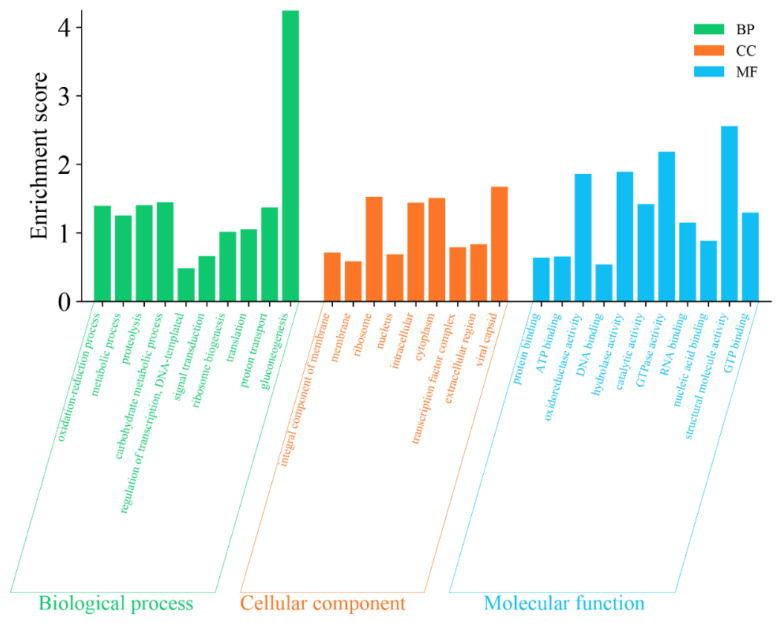
Gene Ontology (GO) term enrichment analysis of differentially abundant proteins (DAPs) of **C** vs. **A**.

The function of the DAPs was annotated using GO analysis and further classified into the categories of MF, CC, and BP. The GO term enrichment analysis of **C** vs. **A** (Figure 6 and Table S6) demonstrated that the main BP involved oxidation reduction process, metabolic process, proteolysis, carbohydrate metabolic process, regulation of transcription, DNA templating, signal transduction, ribosome biogenesis, translation, proton transport, and gluconeogenesis. The main CC involved an integral component of the membrane, membrane, ribosome, nucleus, intracellular, cytoplasm, transcription factor complex, extracellular region, and viral capsid. The main MF involved protein binding, ATP binding, oxidoreductase activity, DNA binding, hydrolase activity, catalytic activity, GTPase activity, RNA binding, nucleic acid binding, structural molecule activity, and GTP binding.

**Figure 7:**
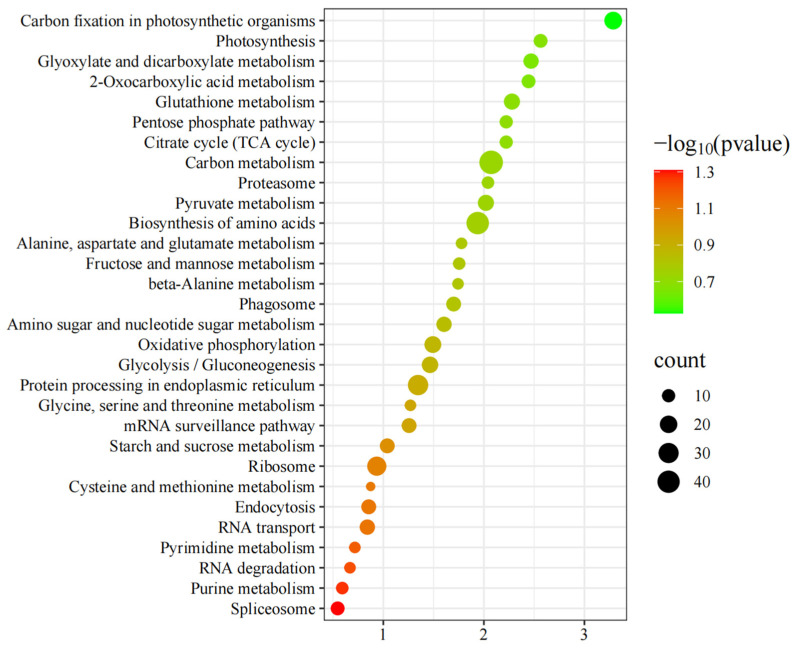
Top twenty-nine Kyoto Encyclopedia of Genes and Genomes (KEGG) enrichment analyses of differentially abundant proteins (DAPs) of **C** vs. **A**.

To further the functional characterization of the DAPs of **C** vs. **A**, a pathway analysis based on the KEGG database was classified and annotated into 129 known KEGG pathways (Table S7). A KEGG pathway analysis of **C** vs. **A** (Figure 7) revealed that DAPs were mainly annotated into Carbon metabolism, Biosynthesis of amino acids, Protein processing in endoplasmic reticulum, Ribosome, Carbon fixation in photosynthetic organisms, Oxidative phosphorylation, Glycolysis/Gluconeogenesis, Pyruvate metabolism, Glutathione metabolism, RNA transport, Amino sugar and nucleotide sugar metabolism, Glyoxylate and dicarboxylate metabolism, Starch and sucrose metabolism, Endocytosis, Phagosome, mRNA surveillance pathway, Photosynthesis, 2-Oxocarboxylic acid metabolism, Spliceosome, Citrate cycle (TCA cycle), Pentose phosphate pathway, Proteasome, Purine metabolism, Fructose and mannose metabolism, Glycine, serine and threonine metabolism, Alanine, aspartate and glutamate metabolism, RNA degradation, beta-Alanine metabolism, Pyrimidine metabolism, and Cysteine and methionine metabolism.

### 3.6. Combined Analysis of Transcriptomic and Proteomic Technology

The combined transcriptome and proteome analysis results show that the DEGs and DAPs of **C** vs. **A** were primarily annotated to the ascorbate and aldarate metabolism (ko00053) pathway, indicating that this metabolic pathway plays a crucial role in influencing AsA accumulation in RRT. As shown in Table 4 and Table S4, a total of 13 DEGs were metabolized for the ascorbate and aldarate metabolism pathway. Meanwhile, as shown in Table 5 and Table S7, a total of 15 DAPs were metabolized for the ascorbate and aldarate metabolism pathway.

## 4. Discussion

Ascorbic acid (AsA), also known as vitamin C, is a crucial antioxidant that is abundantly present in fruits of plants. These fruits, which are rich in AsA, constitute the primary source of this vital nutrient for human consumption [2,3,4,5]. The AsA contents of some fruits are organized, such as *Rosa roxburghii* Tratt (RRT), tomato, kiwifruit, orange, strawberry, carrot, sweet pepper, and so on [32]. RRT is a rare fruit crop cultivated in Southwestern China, and its fruits contain a diverse array of phytochemicals that are beneficial to human health [1]. Notably, the AsA content in RRT is exceptionally high, surpassing that of many common fruits such as tomatoes, strawberries, and kiwifruits [33]. Meanwhile, exogenous AsA treatment can significantly improve the cold resistance of banana fruit and strawberry fruit under low-temperature storage [34,35]. In addition, changes in AsA content are closely associated with fruit ripening. Upon reducing the AsA content in tomato fruits, a significant decrease in fruit size was observed [36]. Therefore, elucidating the AsA contents and underlying mechanisms of AsA accumulation in fruits are crucial for guiding improvements in fruit quality and postharvest preservation.

In this study, the AsA contents in RRT during fruit development were investigated. Our results demonstrate that the AsA content in RRT fruits increased linearly throughout the development process, peaking at 2274.60 mg/(100 g FW) at 84 DAA. Huang et al. also observed comparable trends, noting that AsA initially accumulates during the later stages of RRT development and continues to increase throughout ripening, with the most rapid accumulation occurring near maturity (reaching a peak concentration of 1290 mg/(100 g FW) at 95 DAA) [33]. While several other fruits, including strawberries and tomatoes, exhibit continued AsA accumulation during the latter stages of development [37,38], they do not achieve AsA concentrations as high as those seen in RRT. In contrast, fruits such as acerola, kiwifruit, apple, and peach primarily synthesize AsA during the early stages of development [39,40,41,42].

Because of the important role of AsA in plants, in-depth studies have been carried out on the metabolic pathways of AsA in plants. Studies have found that the main pathways for AsA synthesis in plants are L-galactose pathway, Myoinositol pathway, L-gulose pathway, and D-galacturonate pathway [32]. Our study represents the first investigation into the molecular mechanisms underlying AsA accumulation in RRT during fruit development using transcriptomic and proteomic techniques. The integrated transcriptome and proteome analysis revealed that the DEGs and DAPs between A and C were predominantly annotated to the ascorbate and aldarate metabolism pathway. The ascorbate and aldarate metabolic pathway, which plays a vital role in safeguarding cells against oxidative stress, is intricately linked with the glucuronate pathway [43]. The glucuronic acid generated through this pathway can be utilized for the synthesis of glycosaminoglycans, including heparin, hyaluronic acid, and chondroitin sulfate, and also contributes to ascorbate and aldarate metabolism by providing AsA [44]. Integrating the analysis of metabolome, transcriptome, and physiological by Yin et al. revealed that seven metabolites connected to fructose and mannose metabolism are associated with AsA metabolism during the fruit development of *Lycium chinense* [45]. In an effort to pinpoint candidate genes involved in the AsA metabolism, Yang et al. conducted a transcriptome analysis on *Pugionium cornutum* (L.) Gaertn. leaves under drought conditions using Illumina sequencing. Their study identified and annotated 144 unique genes related to the ascorbate and aldarate metabolic pathway [46].

According to the previous studies, there were significant differences in the expression levels of AsA synthesis and metabolism-related genes in fruits with different AsA contents [47,48]. It is of great significance to improve the AsA content in fruit by changing the expression of GDP-mannose pyrophosphorylase (*GMP*), GDP-D-mannose3′,5′-epimerase (*GME*), GDP-L-galactose-phosphorylase (*GGP*), L-galactono-1,4-lactone dehyd (*GLDH*), L-gulono-γ-lactone oxidase (*GLOase*), myoinositol oxygenase (*MIOX*), D-galacturonate reductase (*GalUR*), ascorbate peroxidase (*APX*), ascorbate oxidase (*AAO*), dehydroascorbate reductase (*DHAR*), and monodehydroascorbate reductase (*MDHAR*) genes related to AsA synthesis and metabolism [32]. In plants, L-ascorbate peroxidase (APX) plays a crucial role in the metabolism of AsA. This enzyme, along with AAO, catalyzes the oxidation of AsA to monodehydroascorbic acid (MDHA) and dehydroascorbic acid (DHA) [49]. L-gulono-1,4-lactone oxidase is not only the last precursor of AsA biosynthesis in L-gulose pathway, but also the last precursor of AsA biosynthesis in the myoinositol pathway [50]. Like in animals, myoinositol can be converted to D-glucuronate by MIOX in plants [51]. It has been reported that overexpression of *AtMIOX4* can increase the AsA content in *Arabidopsis* [52]. Munir et al. identifies five *MIOX* (*MIOX1*-*MIOX5*) genes in tomatoes, and overexpression of the *MIOX4* gene significantly increases the AsA content in leaves and red fruits [5]. As indicated in Table 4 and Table 5, the expression levels of the *APX* (gene IDs: Cluster-181.9935 and Cluster-181.1061), *AAO* (gene ID: Cluster-1826.0), and *MIOX* (gene IDs: Cluster-181.21502) genes were up-regulated, and the protein expression levels of APX (accession ID: XM_004302791), AAO (accession ID: KC782561), and MIOX (accession IDs: XM_004298708 and XM_004297728) were also up-regulated. These findings suggest that the ascorbate and aldarate metabolism pathway plays a critical role in regulating AsA accumulation in RRT.

## 5. Conclusions

In conclusion, our results demonstrate that the AsA content in RRT fruits increased linearly throughout the development process, peaking at 2274.60 mg/(100 g FW) at 84 DAA. Furthermore, integrated transcriptome and proteome analyses revealed that the ascorbate and aldarate metabolism pathway play a crucial role in regulating AsA accumulation in RRT. This study elucidates the molecular mechanisms underlying AsA accumulation in RRT and provides a solid scientific foundation for future research on this topic.

## Figures and Tables

**Table 1:** Overview of transcriptome sequencing date.

Sample ^1^	Number		Error (%)	Q20 (%) ^2^	Q30 (%) ^3^	GC (%) ^4^
	Raw Reads	Clean Reads				
**A-1**	47,491,254	46,202,096	0.03	97.62	93.29	46.28
**A-2**	54,447,622	52,816,246	0.03	97.70	93.50	46.20
**C-1**	43,447,172	42,425,254	0.03	97.69	93.43	46.18
**C-2**	43,119,412	41,712,142	0.03	97.80	93.68	46.25

^1^ Biological replicates for samples **A** and **C**, respectively. ^2^ Base quality > 20. ^3^ Base quality > 30. ^4^ Relative guanine–cytosine content.

**Table 2:** Overview of splicing length and frequency distribution.

Feature	Number				Total Number	Mean Splicing Length
	301–500 bp	500–1000 bp	1000–2000 bp	>2000 bp		
Transcripts	18,279	24,922	33,876	31,134	108,211	1605
Unigenes	8613	9537	9188	9004	36,342	1435

**Table 3:** Overview of the annotation database of transcriptome sequencing date.

Annotation Database	Annotated Number	Percentage (%)
Nt	21,857	60.14
Nr	18,704	51.46
SwissProt	17,322	47.66
PFAM	17,135	47.14
KOG	5633	15.49
KEGG	7905	21.75
GO	17,135	47.14
All databases	2658	7.31
At least one database	26,412	72.67

**Table 4:** DEGs annotated into ascorbate and aldarate metabolism using transcriptomic technology.

Gene ID	Symbol	Gene Name	Trend (Up/Down)
Cluster-4278.2	ALDH	aldehyde dehydrogenase (NAD^+^)	up
Cluster-181.21502	MIOX	myoinositol oxygenase	up
Cluster-181.14069	ALDH	aldehyde dehydrogenase (NAD^+^)	up
Cluster-181.7230	ALDH	aldehyde dehydrogenase (NAD^+^)	up
Cluster-1826.0	AAO	L-ascorbate oxidase	up
Cluster-181.10833	GME	GDP-D-mannose 3′,5′-epimerase	up
Cluster-181.9935	APX	L-ascorbate peroxidase	up
Cluster-181.6123	VTC2_5	GDP-L-galactose phosphorylase	up
Cluster-181.1061	APX	L-ascorbate peroxidase	up
Cluster-181.10457	APX	L-ascorbate peroxidase	down
Cluster-181.10851	VTC2_5	GDP-L-galactose phosphorylase	down
Cluster-181.688	UGDH, ugd	UDP-glucose 6-dehydrogenase	down
Cluster-181.21647	MIOX	myoinositol oxygenase	down

**Table 5:** DAPs annotated into ascorbate and aldarate metabolism using proteomic technology.

Accession ID	Protein Name	Trend (Up/Down)
XM_009155117	Exophiala dermatitidis NIH/UT8656 aldehyde dehydrogenase partial mRNA	up
XM_004302791	PREDICTED: Fragaria vesca subsp. vesca L-ascorbate peroxidase 2, cytosolic-like (LOC101298335), mRNA	up
KC782561	Rosa roxburghii isolate AO1680bp ascorbate oxidase mRNA, partial cds	up
XM_004298708	PREDICTED: Fragaria vesca subsp. vesca myoinositol oxygenase 1-like (LOC101293781), mRNA	up
XM_004297728	PREDICTED: Fragaria vesca subsp. vesca myoinositol oxygenase 1-like (LOC101302448), mRNA	up
XM_004299270	PREDICTED: Fragaria vesca subsp. vesca UDP-glucose 6-dehydrogenase 5 (LOC101292899), transcript variant X1, mRNA	up
XM_011469911	PREDICTED: Fragaria vesca subsp. vesca aldehyde dehydrogenase family 3 member H1-like (LOC101313059), transcript variant X3, mRNA	down
XM_016906917	Sphaerulina musiva SO2202 aldehyde dehydrogenase mRNA	down
XM_011460875	PREDICTED: Fragaria vesca subsp. vesca putative L-ascorbate peroxidase 6 (LOC101295782), mRNA	down
XM_004294353	PREDICTED: Fragaria vesca subsp. vesca GDP-L-galactose phosphorylase 2 (LOC101314355), mRNA	down
XM_004293935	PREDICTED: Fragaria vesca subsp. vesca GDP-L-galactose phosphorylase 1-like (LOC101307284), transcript variant X1, mRNA	down
KC782575	Rosa roxburghii isolate GME GDP-D-mannose-3′,5′-epimerase mRNA, complete cds	down
XM_007683098	Baudoinia panamericana UAMH 10762 hypothetical protein mRNA	down
XM_013571415	Aureobasidium namibiae CBS 147.97 aldehyde dehydrogenase, allergen Cla h 10 partial mRNA	down
XM_004299625	PREDICTED: Fragaria vesca subsp. vesca thylakoid lumenal 29 kDa protein, chloroplastic (LOC101302838), mRNA	down
XM_011469911	PREDICTED: Fragaria vesca subsp. vesca aldehyde dehydrogenase family 3 member H1-like (LOC101313059), transcript variant X3, mRNA	down

## Data Availability

The raw data supporting the conclusions of this article will be made available by the authors upon request. The raw data of proteome were all deposited at http://www.proteomexchange.org/ (accessed on 10 October 2024) with the accession project ID: PXD056713.

## Supplementary Materials

The following supporting information can be downloaded at: https://www.mdpi.com/article/10.3390/foods14050748/s1, Table S1. The total cDNA library prepared from RRT. Table S2. List of DEGs of **C** vs. **A**. Table S3. The classified and annotated GO terms of DEGs of **C** vs. **A**. Table S4. KEGG pathways enrichment of DEGs of **C** vs. **A**. Table S5. List of the total proteins of **C** vs. **A**. Table S6. The classified and annotated GO terms of DAPs of **C** vs. **A**. Table S7. KEGG pathways enrichment of DAPs of **C** vs. **A**.
